# Supplementary material for: Hic-5 drives epithelial mechanotransduction promoting a feed-forward cycle of bronchoconstriction
Source: Nat Commun. 2025 Dec 12;17:516. doi: 10.1038/s41467-025-67210-9 (PMC12804887; doi:10.1038/s41467-025-67210-9)
Supplement: Supplementary file 1 — Supplementary Information [file 41467_2025_67210_MOESM1_ESM.pdf]

Supplementary information

Hic-5 drives epithelial mechanotransduction promoting a feed-forward cycle of bronchoconstriction.

Chimwemwe Mwase[1], Wenjiang Deng[1], Hyo Jin Kim[1], Jennifer A. Mitchel[1], Thien-Khoi Phung[1], Michael J. O'Sullivan[1], Joel A. Mathews[2], Jeffrey Crosby[2], Christopher E. Turner[3], Adam L. Haber[1], Jin-Ah Park[1]

[1] Department of Environmental Health, Harvard T.H. Chan School of Public Health, Boston, MA, USA.

[2] Ionis Pharmaceuticals, Carlsbad, CA, USA

[3] Department of Cell and Developmental Biology, SUNY Upstate Medical University, Syracuse, NY, USA

Corresponding author:

Jin-Ah Park, PhD

Department of Environmental Health

Harvard T.H. Chan School of Public Health

665 Huntington Ave, SPH1-315

Boston, MA 02115

Email: jpark@hsph.harvard.edu

Supplementary Figures

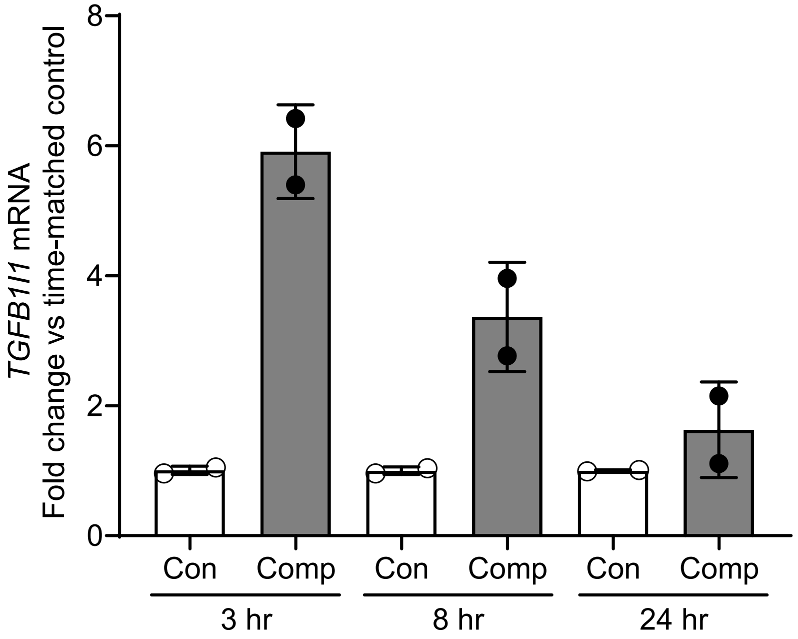

Supplementary Figure 1. Time-dependent induction of *TGFB1I1* mRNA expression following mechanical compression in HBE cells.
TGFB1I1 mRNA expression was measured by qPCR at the indicated time points (3, 8, and 24 hours) after mechanical compression. *TGFB1I1* mRNA expression was significantly increased at 3 hours and 8 hours post-compression. Data are presented as fold change relative to time-matched controls (mean ± SD, n = 2 transwells in a single HBE cell donor). Each symbol represents an individual transwell: open circles for control and closed circles for compression

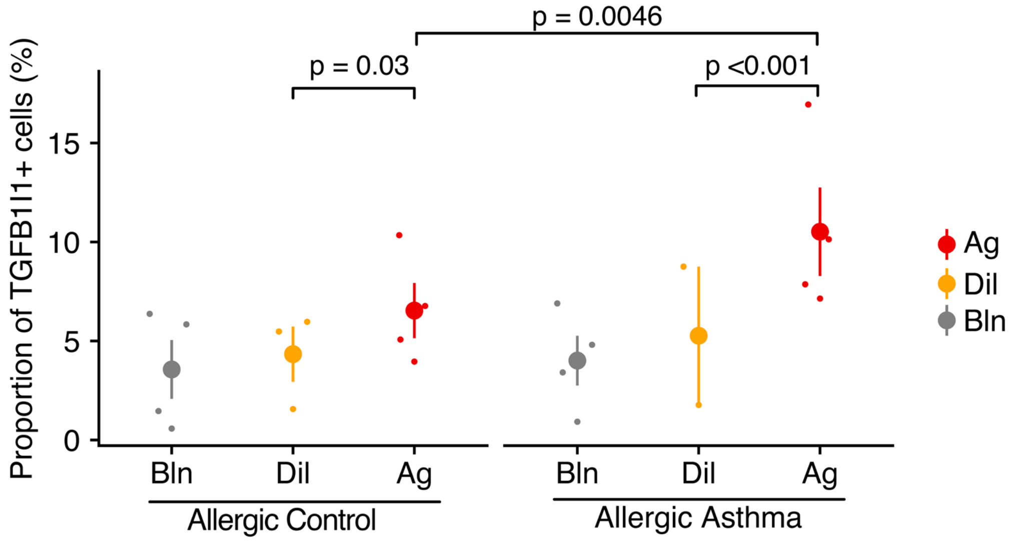

Supplementary Figure 2. Allergen challenge significantly induced *TGFB1I1* expression, with a significantly greater induction in subjects with allergic asthma than in allergic controls.
Each small dot represents individual patients under the conditions indicated on the X-axis. Large dots represent the mean of individual human donors, mean ± SEM (n=4 patients). *P*-value: MAST likelihood-ratio test, two-sided.

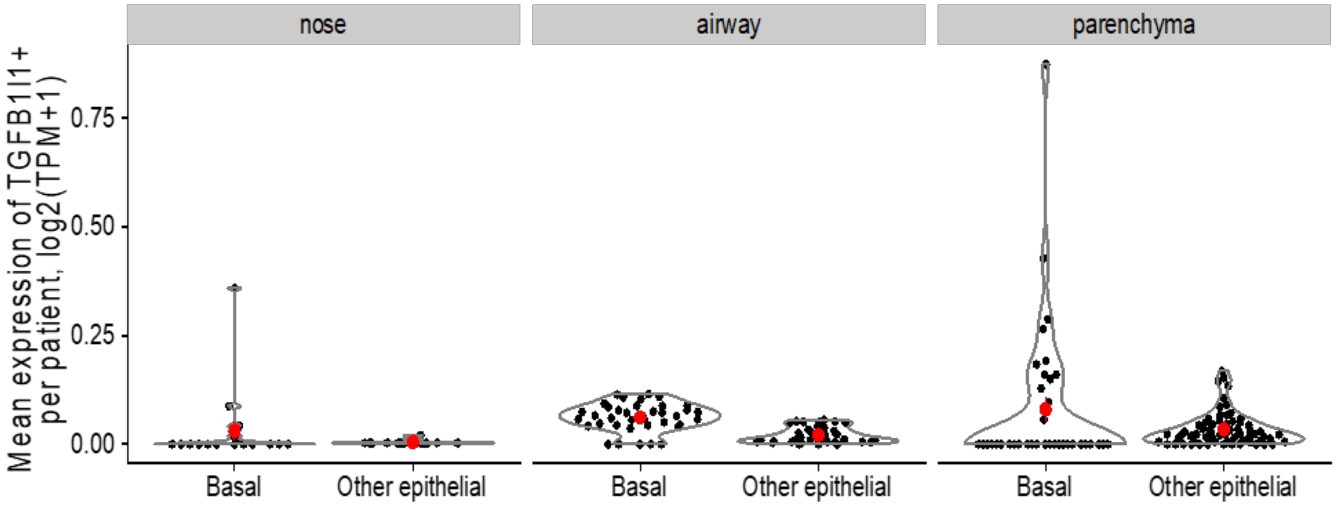

Supplementary Figure 3. Re-analysis of published single-cell RNA-seq (scRNA-seq) data[17] reveals the spatial distribution of *TGFB1I1* expression.

Violin plots show *TGFB1I1* expression levels in basal vs non-basal (other) epithelial cells across the respiratory tract, including the nose, airways, and lung parenchyma. In the nose, *TGFB1I1* expression appeared to be absent in most donors. In both airways and parenchyma, *TGFB1I1* expression was greater in basal cells compared to non-basal cells. Each black dot represents each human donor. Each closed dot represents individual human donor, and the red dot represents the mean value in the indicated cell types.

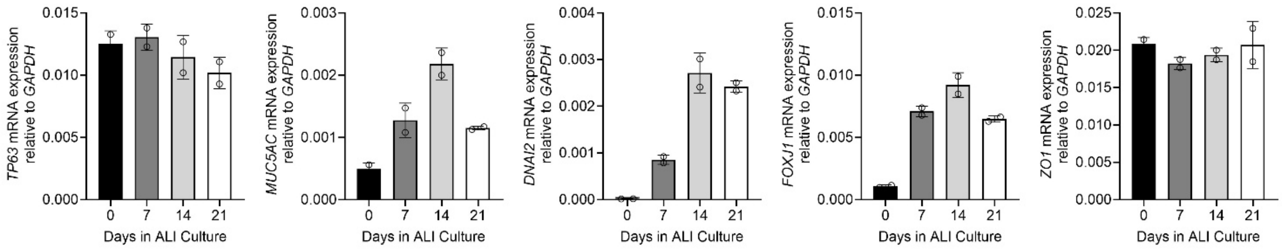

Supplementary Figure 4. Primary human bronchial epithelial (HBE) cells progressively differentiate over the course of 21 days in air–liquid interface (ALI) culture, as assessed by epithelial cell-type specific markers.

During ALI culture, expression of *MUC5AC* for goblet cells and *FOXJ1* and *DNAI2* for ciliated cells progressively increased, indicating proper cellular differentiation. The expression of T*P63* for basal cells and *ZO1* for tight junctions remained stable during differentiation. The expression of each gene is presented relative to *GAPDH* for each ALI day. Data are represented as mean ± SD (n=2 transwells in a single HBE cell donor).

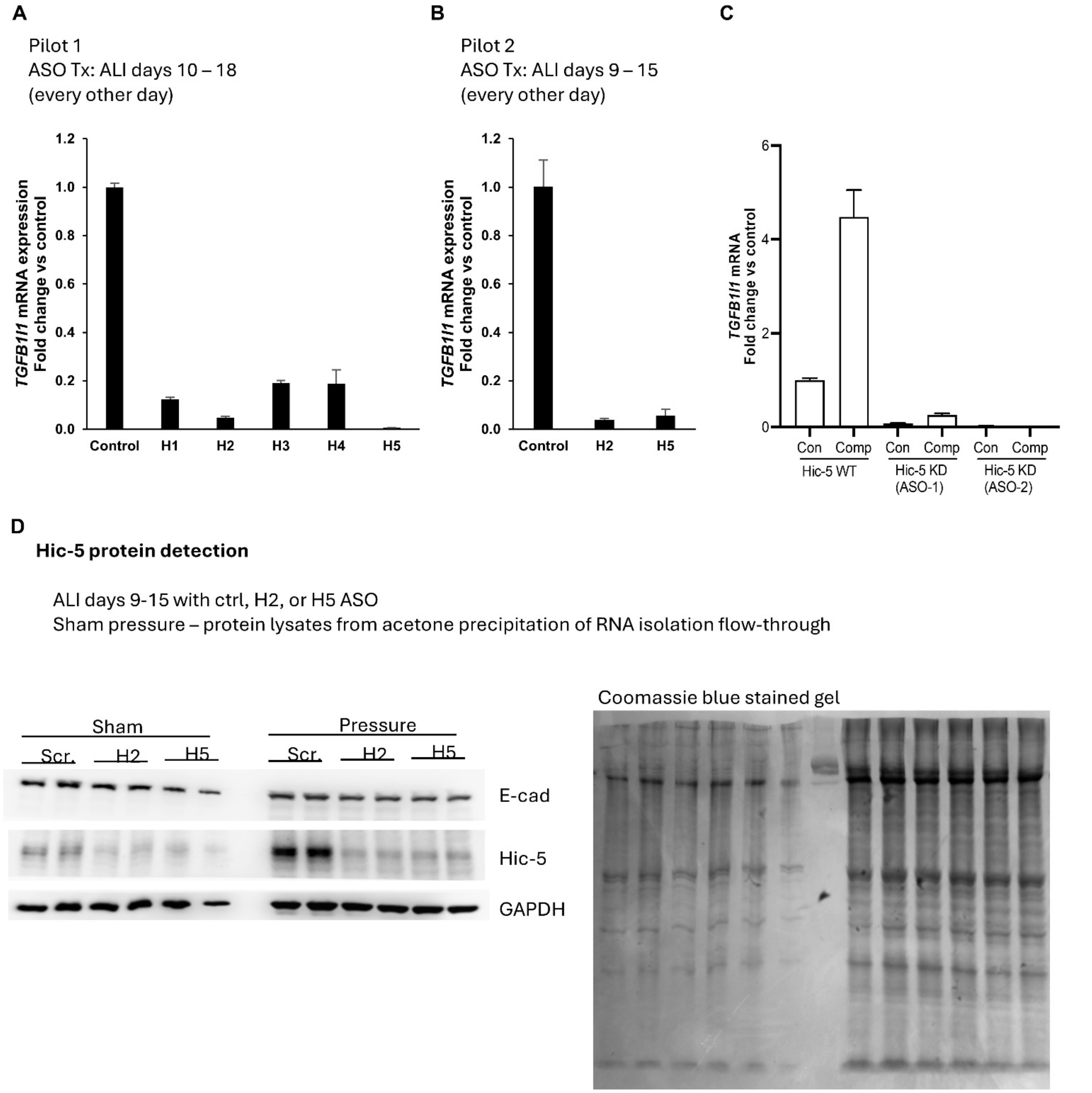

Supplementary Figure 5. Antisense oligonucleotide (ASO) generates effective Hic-5 knockdown (KD) in well-differentiated primary HBE cells in ALI culture.

During optimization of ASO-mediated Hic-5 KD, *TGFB1I1* mRNA expression **(A-C)** and Hic-5 protein **(D)** were measured in well-differentiated HBE cells (n = 3 HBE cell donors). Five sets of antisense oligonucleotides (annotated as H1–H5) targeting Hic-5 was tested in HBE cells without compression **(A and B).** Then, based on the efficacy, H2 (ASO-1) and H5 (ASO-2) were selected further testing in HBE cells with and without compression **(C-D)**. Both H2 and H5 established effective Hic-5 KD, as measured by qPCR **(C)** and western blot analysis **(D)**.

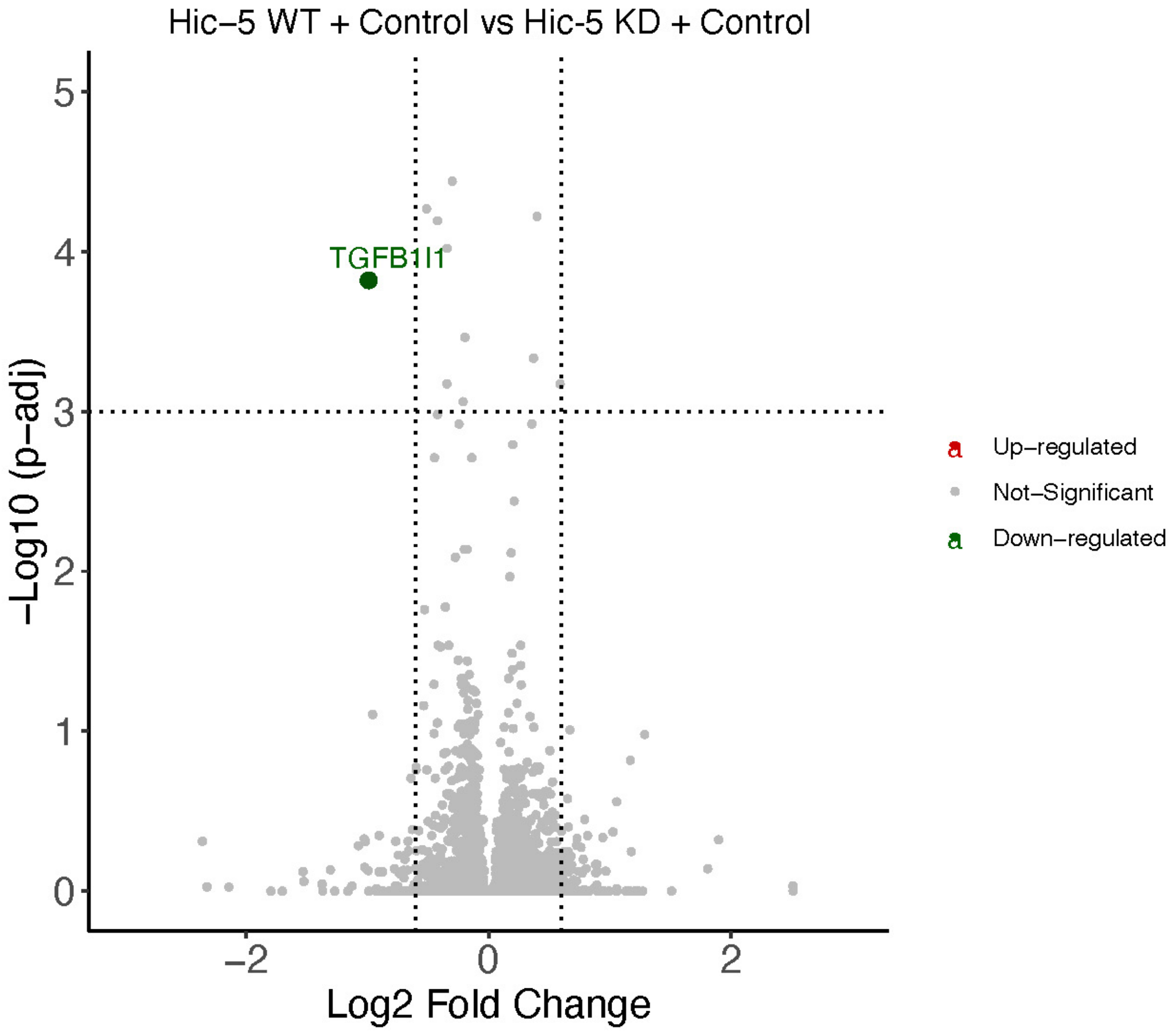

Supplementary Figure 6. The Volcano plot presents differentially expressed genes (DEGs) between Hic-5 WT and KD HBE cells under control conditions. Only Hic-5 was significantly reduced in Hic-5 KD HBE cells.

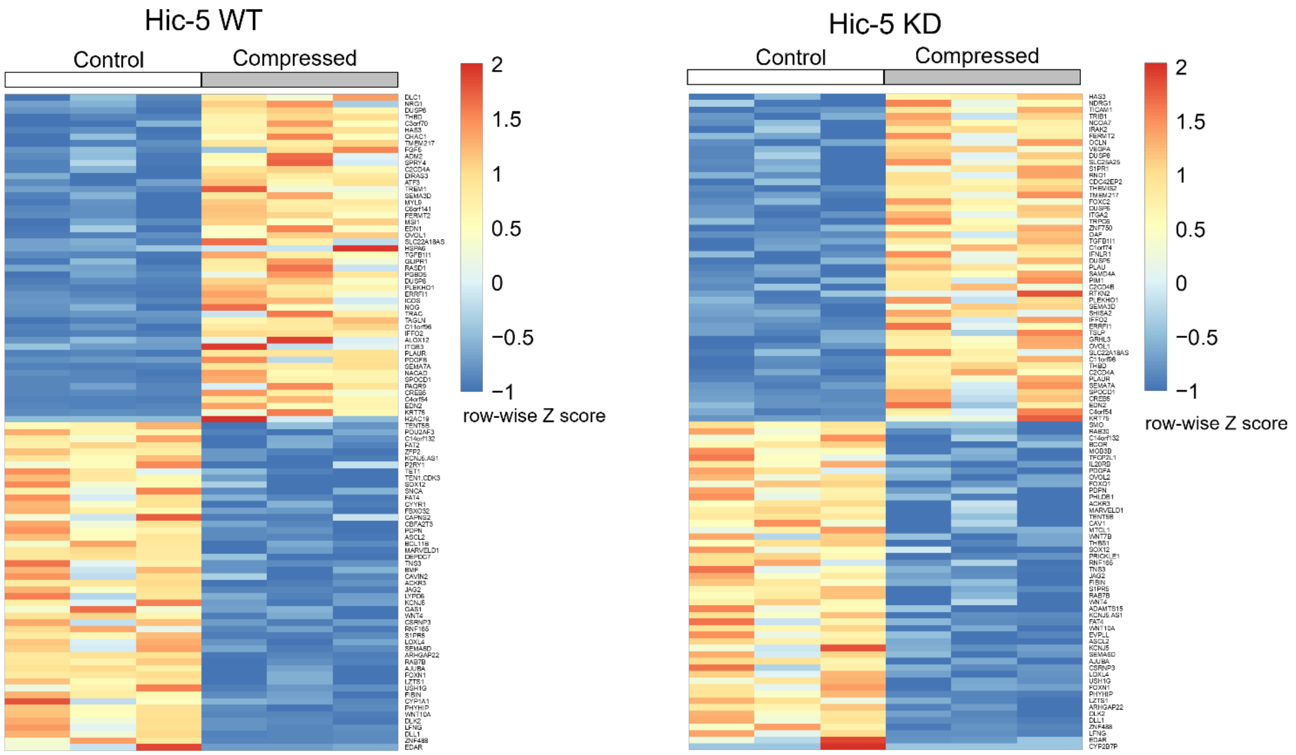

Supplementary Figure 7. Heatmaps present differentially regulated mechanoresponsive genes in Hic-5 WT and Hic-5 KD HBE cells in response to mechanical compression.
Heatmaps show the differentially expressed genes in HBE cells between control and compressed conditions. Left panel: Hic-5 WT cells; right panel: Hic-5 KD cells. Each row represents a denoted gene, and expression values are presented as row-wise Z scores.

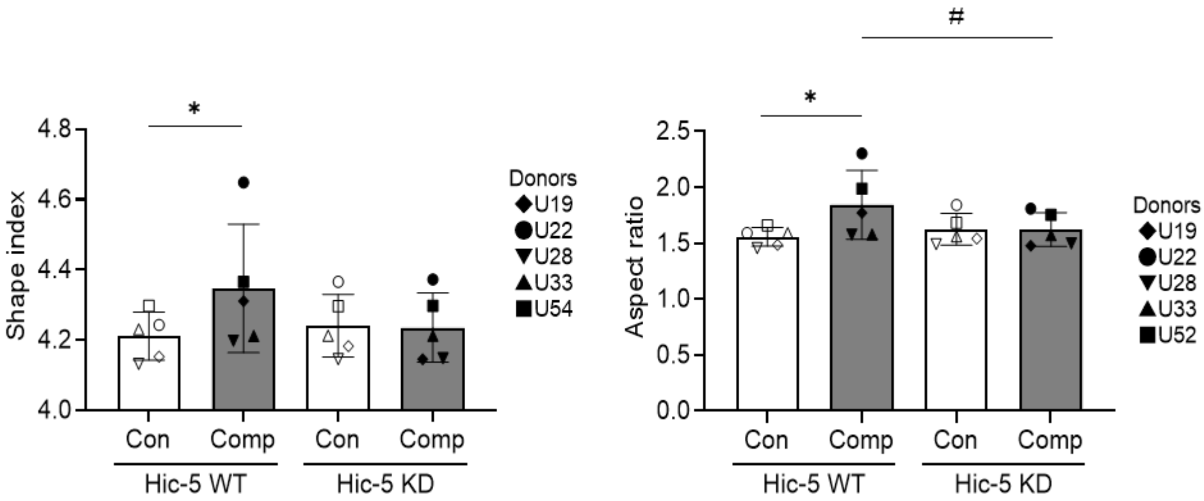

Supplementary Figure 8. Cell shape index (A) and aspect ratio (B) indicate that compression-induced apical cell elongation was attenuated in Hic-5 KD cells. Data are represented as mean ± SD (n=5 HBE cell donors). $^{*,\#}p < 0.05$ vs. WT-Control, two-way ANOVA with Holm–Šídák post-hoc correction. Each symbol represents an individual donor: open symbols for control and closed symbols for compression.

Supplementary Tables

Supplementary Table 1. Demographics of human donors used for isolating primary HBE cells.

Donor No.	Sex	Age
U3	M	28
U7	F	16
U8	F	23
U9	F	59
U10	F	39
U13	M	50
U16	F	47
U17	M	43
U19	M	70
U22	F	32
U23	M	21
U26	F	20
U28	M	59
U30	M	35
U33	M	60
U47	M	17
U56	F	52

Supplementary Table 2. Primer sequences used in RT-qPCR

Gene	Primers	Reference
GAPDH	FW 5'-TGGGCTACACTGAGCACCAG-3'<br>RV 5'-GGGTGTCGCTGTTGAAGTCA-3'	4
EDN1	FW 5'-AGAGTGTGTCTACTTCTGCCA-3'<br>RV 5'-CTTCCAAGTCCATACGGAACAA-3'	5
TGFB1I1	FW 5'- GACTTCCTGCAGCTGTTCG-3'<br>RV 5'- AAGTGGTTCTCGCACAACG-3'	10
PDGFB	FW 5'- ACTCGATCCGCTCCTTTGATGA-3'<br>RV 5'- GCTCGCCTCCAGAGTGGG-3'	11
