# Supplementary material for: Hic-5 drives epithelial mechanotransduction promoting a feed-forward cycle of bronchoconstriction
Source: Nat Commun. 2025 Dec 12;17:516. doi: 10.1038/s41467-025-67210-9 (PMC12804887; doi:10.1038/s41467-025-67210-9)
Supplement: Supplementary file 2 — Reporting Summary [file 41467_2025_67210_MOESM2_ESM.pdf]

nature portfolio

Corresponding author(s): Jin-Ah Park

Last updated by author(s): Oct 30, 2025

Reporting Summary

Nature Portfolio wishes to improve the reproducibility of the work that we publish. This form provides structure for consistency and transparency in reporting. For further information on Nature Portfolio policies, see our Editorial Policies and the Editorial Policy Checklist.

Statistics

For all statistical analyses, confirm that the following items are present in the figure legend, table legend, main text, or Methods section.

n/a	Confirmed	
☐	☒	The exact sample size (*n*) for each experimental group/condition, given as a discrete number and unit of measurement
☐	☒	A statement on whether measurements were taken from distinct samples or whether the same sample was measured repeatedly
☐	☒	The statistical test(s) used AND whether they are one- or two-sided<br>*Only common tests should be described solely by name; describe more complex techniques in the Methods section.*
☐	☒	A description of all covariates tested
☐	☒	A description of any assumptions or corrections, such as tests of normality and adjustment for multiple comparisons
☐	☒	A full description of the statistical parameters including central tendency (e.g. means) or other basic estimates (e.g. regression coefficient) AND variation (e.g. standard deviation) or associated estimates of uncertainty (e.g. confidence intervals)
☐	☒	For null hypothesis testing, the test statistic (e.g. *F*, *t*, *r*) with confidence intervals, effect sizes, degrees of freedom and *P* value noted<br>*Give P values as exact values whenever suitable.*
☒	☐	For Bayesian analysis, information on the choice of priors and Markov chain Monte Carlo settings
☒	☐	For hierarchical and complex designs, identification of the appropriate level for tests and full reporting of outcomes
☒	☐	Estimates of effect sizes (e.g. Cohen's *d*, Pearson's *r*), indicating how they were calculated

Our web collection on statistics for biologists contains articles on many of the points above.

Software and code

Policy information about availability of computer code

Data collection	Blot images were captured using Syngene GeneSys software (v1.7.9; Syngene, Cambridge, UK) and IF images were processed using Zeiss ZEN software (blue edition, v3.3). Both blots and IF images were anlalyzed using ImageJ software (v1.46; National Institutes of Health, Bethesda, MD, USA).
Data analysis	All the data, except the RNA seq data, were analyzed using GraphPad Prism.

For manuscripts utilizing custom algorithms or software that are central to the research but not yet described in published literature, software must be made available to editors and reviewers. We strongly encourage code deposition in a community repository (e.g. GitHub). See the Nature Portfolio guidelines for submitting code & software for further information.

Data

Policy information about availability of data

All manuscripts must include a data availability statement. This statement should provide the following information, where applicable:

- Accession codes, unique identifiers, or web links for publicly available datasets
- A description of any restrictions on data availability
- For clinical datasets or third party data, please ensure that the statement adheres to our policy

Data that comprise the graphs within this manuscript and other findings of this study are available from the corresponding author upon request.

Research involving human participants, their data, or biological material

Policy information about studies with human participants or human data. See also policy information about sex, gender (identity/presentation), and sexual orientation and race, ethnicity and racism.

Reporting on sex and gender	Sex- or gender-based analyses were not performed.
Reporting on race, ethnicity, or other socially relevant groupings	Race and ethnicity information was not reported, as it was not considered relevant to the study objectives.
Population characteristics	Primary human bronchial epithelial (HBE) cells were obtained from thirteen distinct donors with no history of smoking or lung disease.
Recruitment	Cells were obtained from the Marsico Lung Institute/Cystic Fibrosis Center at the University of North Carolina, Chapel Hill, and were originally collected under IRB-approved protocol No. 03-1396.
Ethics oversight	The original collection of the primary HBE cells was conducted under protocol No. 03-1396, approved by the University of North Carolina Biomedical Institutional Review Board.

Note that full information on the approval of the study protocol must also be provided in the manuscript.

Field-specific reporting

Please select the one below that is the best fit for your research. If you are not sure, read the appropriate sections before making your selection.

☒ Life sciences ☐ Behavioural & social sciences ☐ Ecological, evolutionary & environmental sciences

For a reference copy of the document with all sections, see nature.com/documents/nr-reporting-summary-flat.pdf

Life sciences study design

All studies must disclose on these points even when the disclosure is negative.

Sample size	Sample size was not determined using statistical power calculations. The number of donors used in each experiment was based on availability of primary HBE cells meeting inclusion criteria (non-smoking, no lung disease), and aligned with previously established standards in the field. A minimum of three independent donors (except Fig. 2A) was used per experiment to ensure biological reproducibility.
Data exclusions	No data were excluded from the analyses. All samples and conditions included in the study were analyzed and reported.
Replication	All key experiments were independently replicated using primary HBE cells from randomly selected donors. Experiments were performed by three different investigators, and reproducible results were obtained across replicates and operators.
Randomization	Primary HBE cells were cultured from donors who met the inclusion criteria (non-smoking and no history of lung disease), and donors were randomly selected within this defined pool. All experimental conditions were applied to cells from the same donor to control for inter-donor variability.
Blinding	Experiments were performed by different experimenters who were blinded to both the group assignments and expected outcomes.

Reporting for specific materials, systems and methods

We require information from authors about some types of materials, experimental systems and methods used in many studies. Here, indicate whether each material, system or method listed is relevant to your study. If you are not sure if a list item applies to your research, read the appropriate section before selecting a response.

Materials & experimental systems

n/a	Involved in the study	
☐	☒	Antibodies
☐	☒	Eukaryotic cell lines
☒	☐	Palaeontology and archaeology
☒	☐	Animals and other organisms
☒	☐	Clinical data
☒	☐	Dual use research of concern
☒	☐	Plants

Methods

n/a	Involved in the study	
☒	☐	ChIP-seq
☒	☐	Flow cytometry
☒	☐	MRI-based neuroimaging

Antibodies

Antibodies used	The following primary antibodies were used in this study:<br>Hic-5 (BD Biosciences, Cat# 611164, mouse monoclonal), GAPDH (Cell Signaling Technology, Cat# 5174S, rabbit monoclonal), E-cadherin (Cell Signaling Technology, Cat# 3195, rabbit monoclonal at 1:1000), p-ERK (Cell Signaling Technology, Cat# 4370S, rabbit monoclonal, at 1:1000)<br>p-SMAD2 (Cell Signaling Technology, Cat# 3108, rabbit monoclonal, at 1:1000), p-SMAD3 (Cell Signaling Technology, Cat# 9520, rabbit monoclonal, at 1:1000), Antibodies were used for western blotting as described in the Materials and Methods section.
Validation	All antibodies were commercially sourced and validated by the manufacturers for human protein detection in western blot applications. Validation details, including target specificity and tested applications, are available on the manufacturers' websites.

Eukaryotic cell lines

Policy information about cell lines and Sex and Gender in Research

Cell line source(s)	We used primary human bronchial epithelial (HBE) cells isolated at the Marsico Lung Institute/Cystic Fibrosis Research Center, the University of North Carolina (UNC), Chapel Hill. Donor lungs were obtained under a protocol approved by the UNC Institutional Review Board (approval no. 03-1396). Informed consent was obtained from the legally authorized representatives of all organ donors. This has been included in the ethics statement in the Method section.
Authentication	*Describe the authentication procedures for each cell line used OR declare that none of the cell lines used were authenticated.*
Mycoplasma contamination	*Confirm that all cell lines tested negative for mycoplasma contamination OR describe the results of the testing for mycoplasma contamination OR declare that the cell lines were not tested for mycoplasma contamination.*
Commonly misidentified lines<br>(See ICLAC register)	*Name any commonly misidentified cell lines used in the study and provide a rationale for their use.*

Plants

Seed stocks	*Report on the source of all seed stocks or other plant material used. If applicable, state the seed stock centre and catalogue number. If plant specimens were collected from the field, describe the collection location, date and sampling procedures.*
Novel plant genotypes	*Describe the methods by which all novel plant genotypes were produced. This includes those generated by transgenic approaches, gene editing, chemical/radiation-based mutagenesis and hybridization. For transgenic lines, describe the transformation method, the number of independent lines analyzed and the generation upon which experiments were performed. For gene-edited lines, describe the editor used, the endogenous sequence targeted for editing, the targeting guide RNA sequence (if applicable) and how the editor was applied.*
Authentication	*Describe any authentication procedures for each seed stock used or novel genotype generated. Describe any experiments used to assess the effect of a mutation and, where applicable, how potential secondary effects (e.g. second site T-DNA insertions, mosiacism, off-target gene editing) were examined.*
