# Supplementary figures and images for: Hic-5 drives epithelial mechanotransduction promoting a feed-forward cycle of bronchoconstriction
Source: Nat Commun. 2025 Dec 12;17:516. doi: 10.1038/s41467-025-67210-9 (PMC12804887; doi:10.1038/s41467-025-67210-9)

Figure 2C

U19

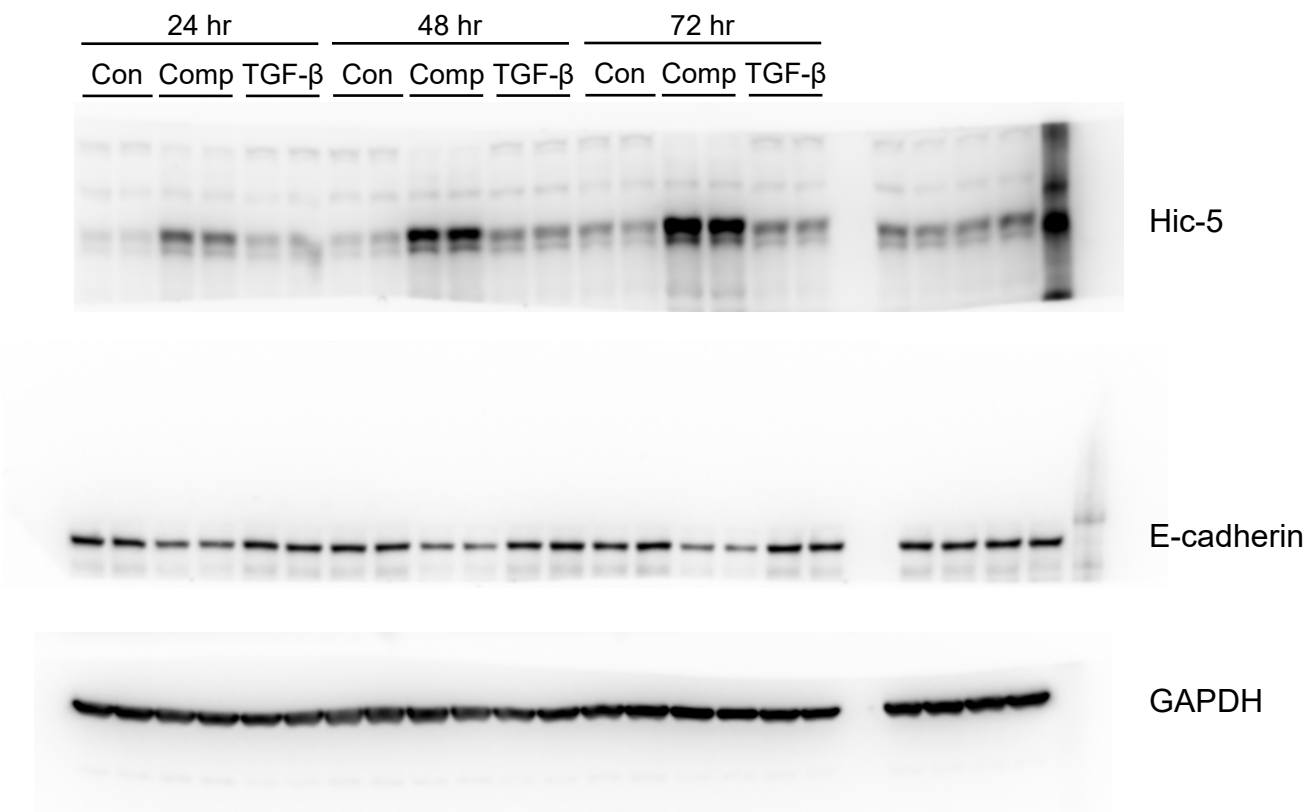

### Figure 2C

U8

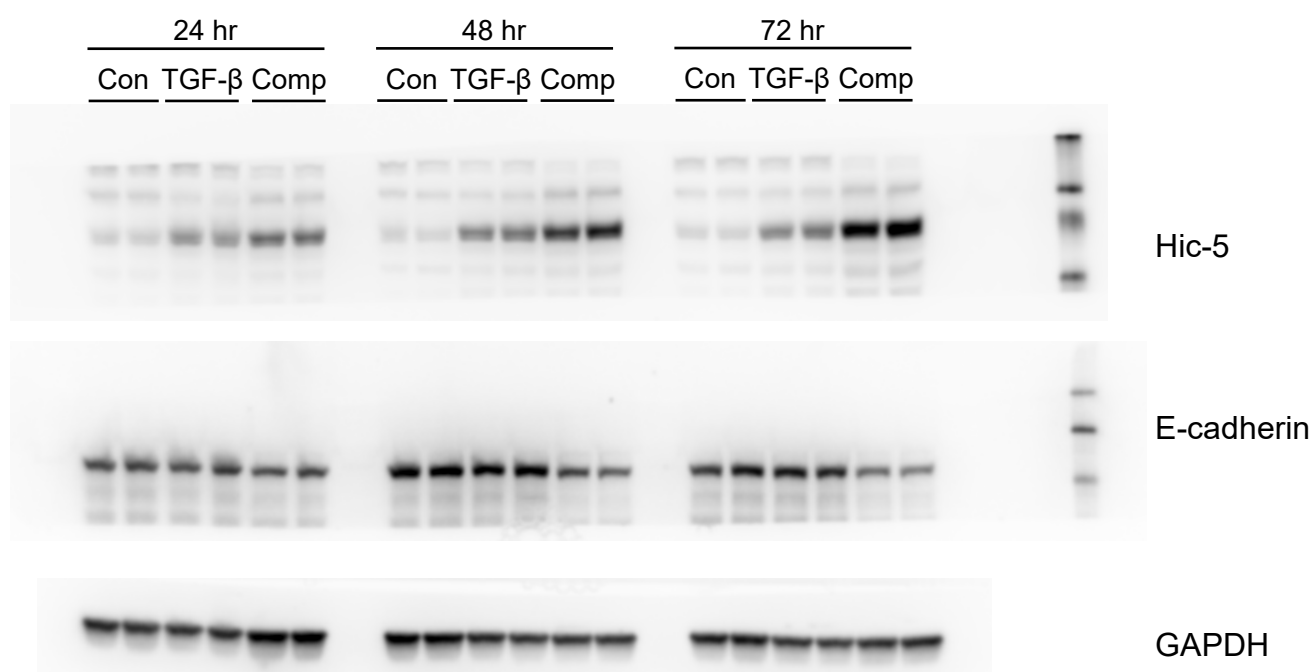

Figure 2D

U17

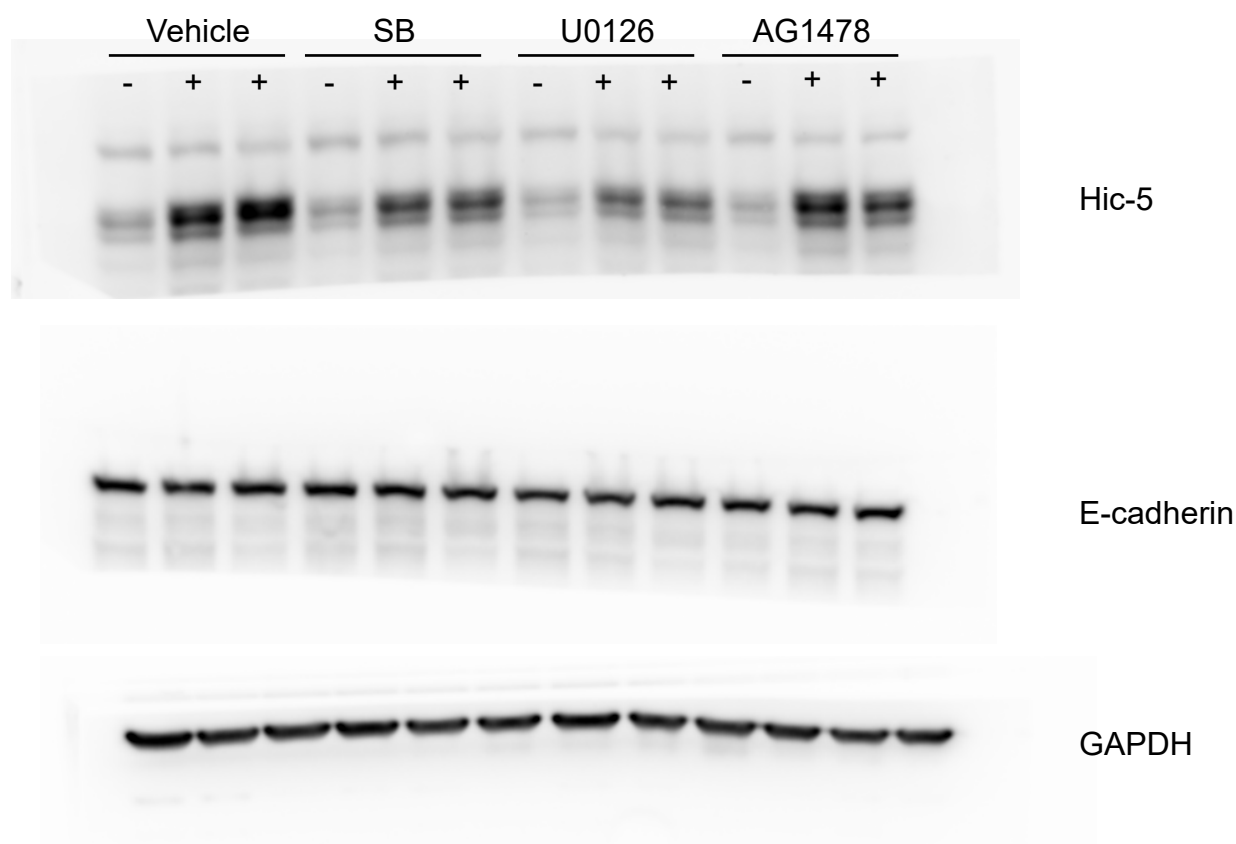

Figure 2D

U16

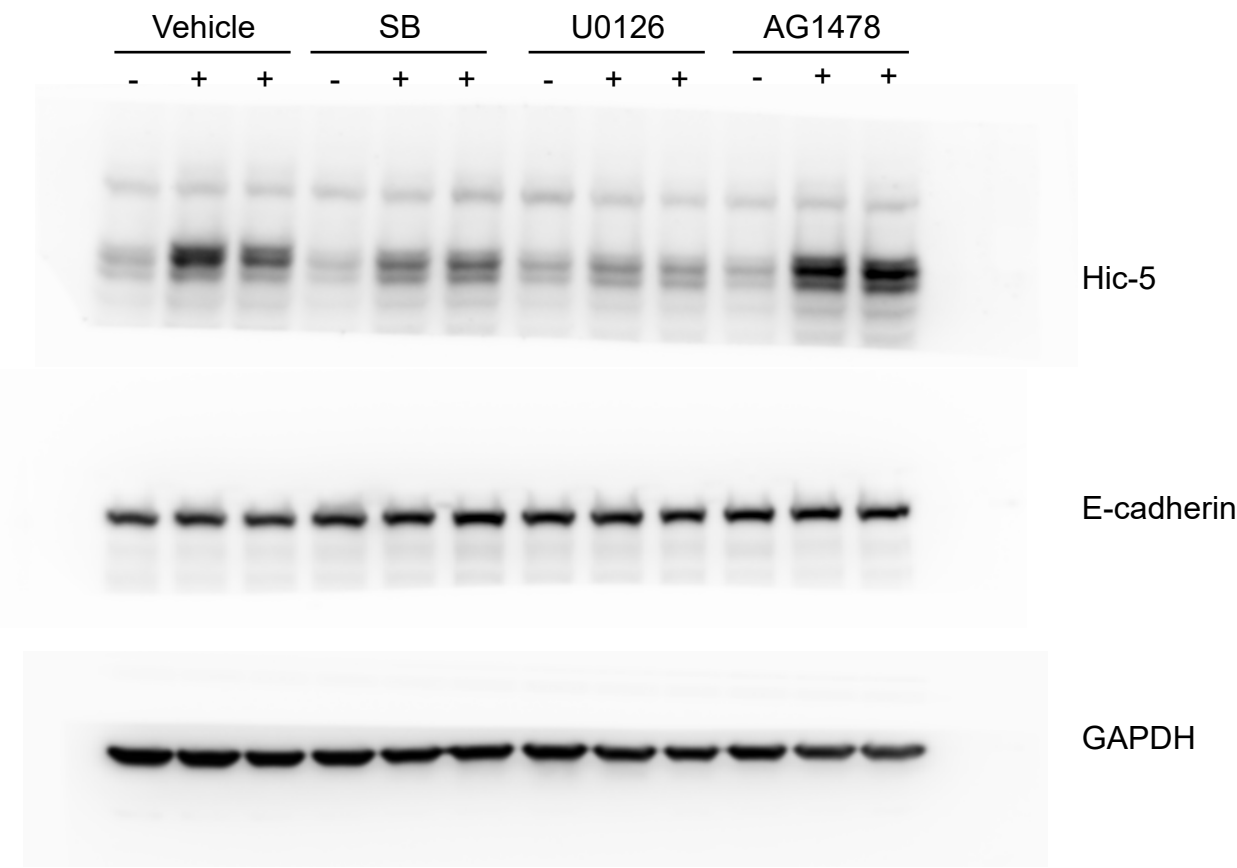

Figure 2E

U3

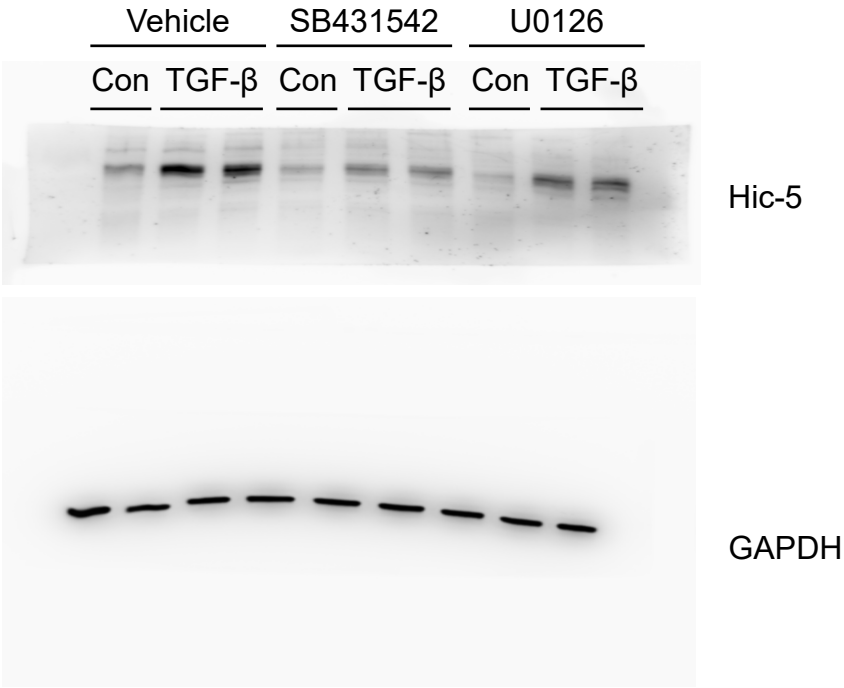

U22

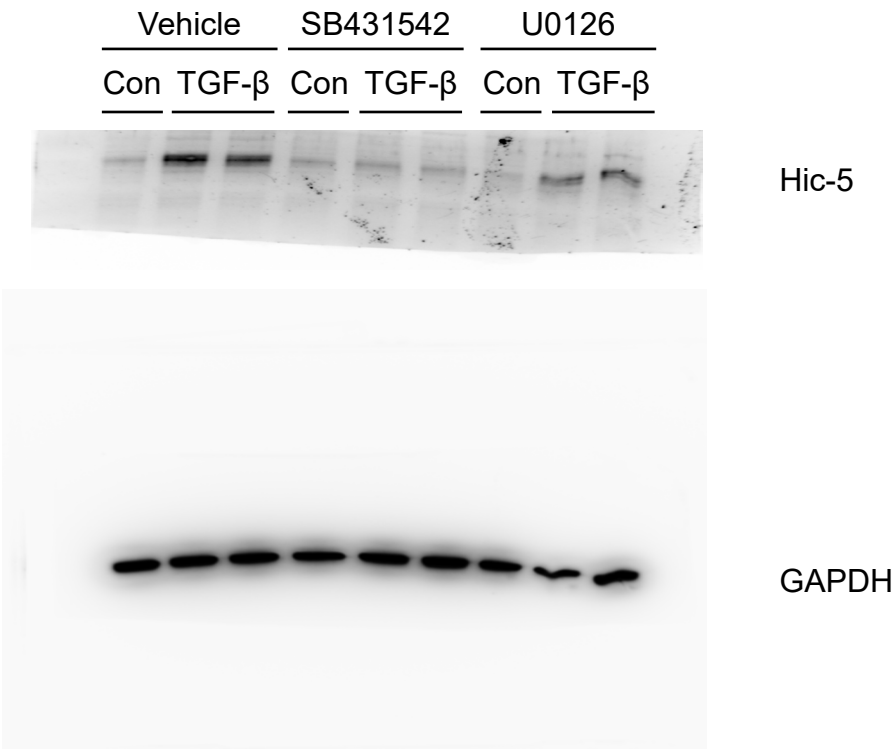

Figure 3A

U19

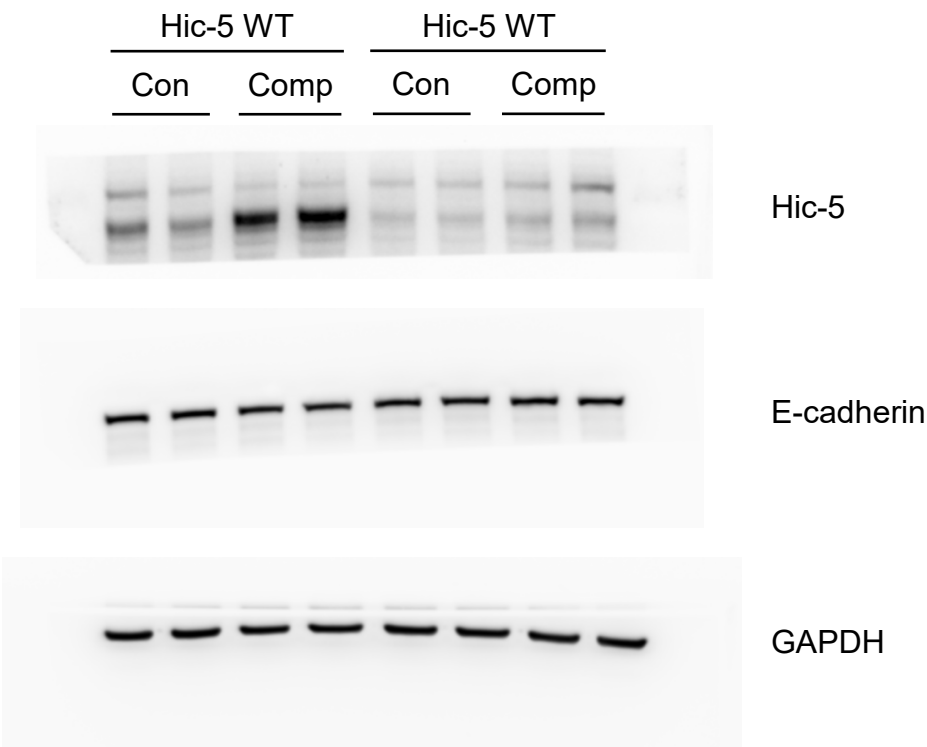

U26

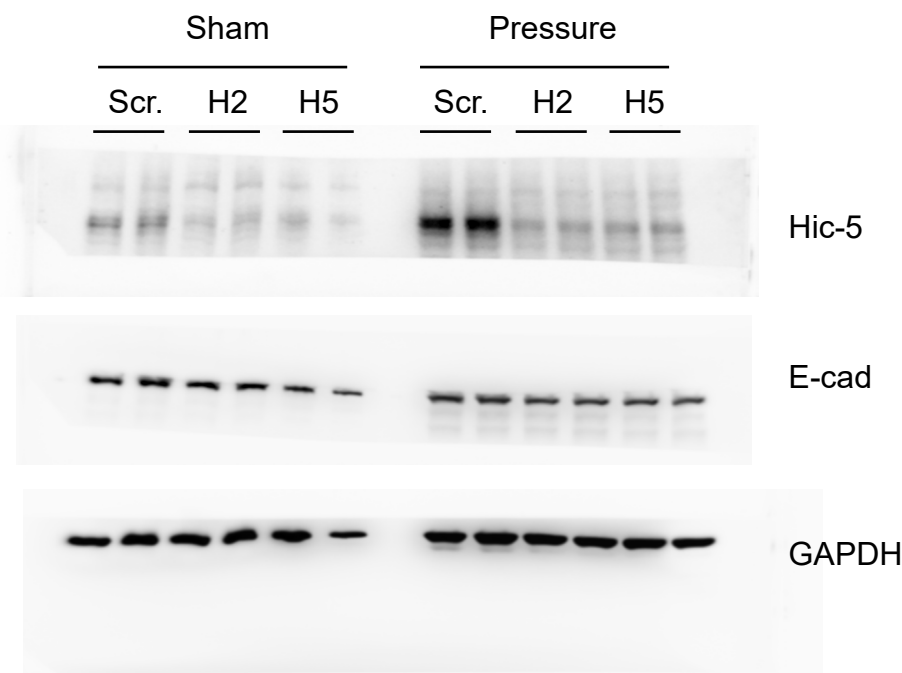

Figure 3A

U30

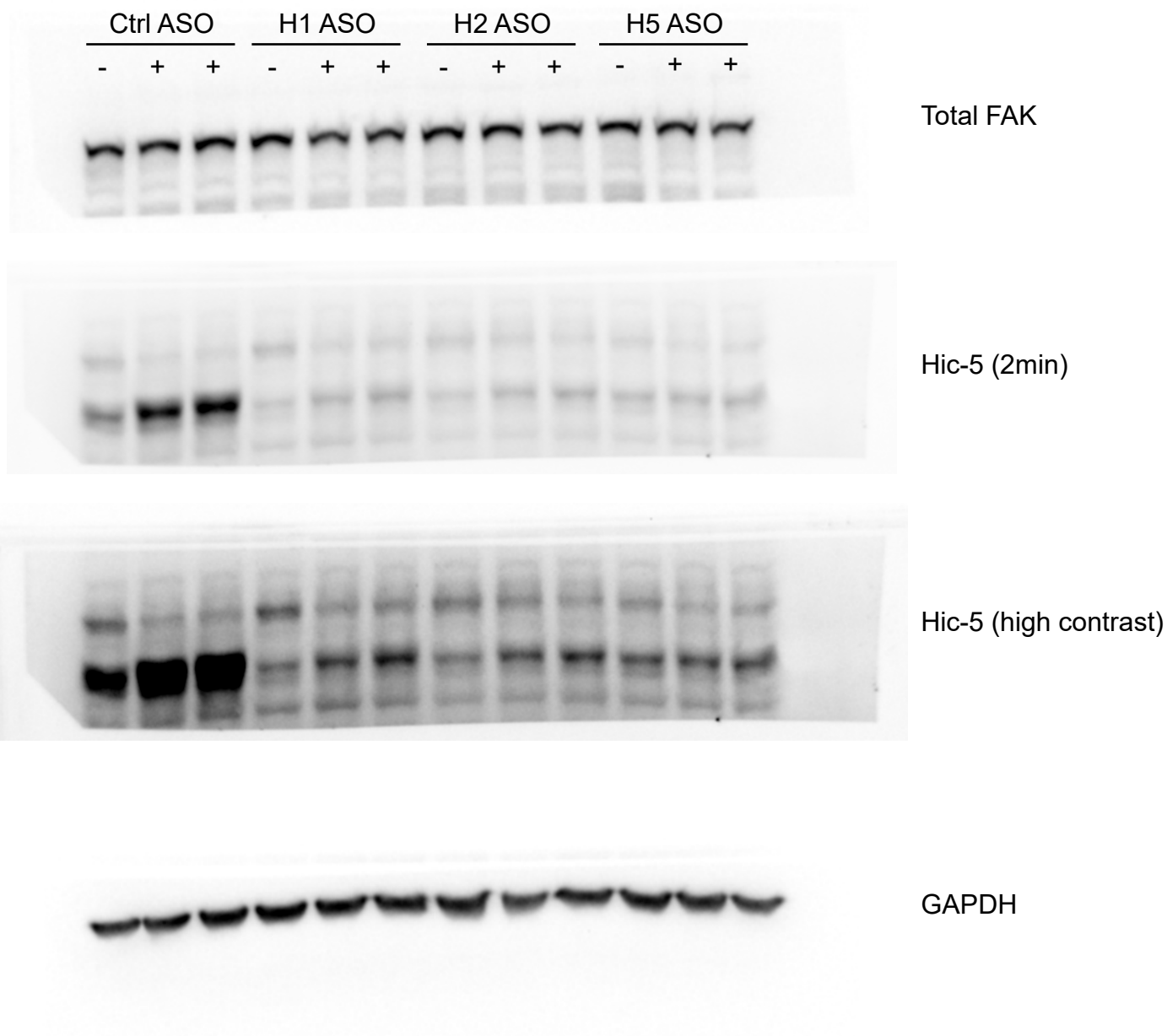

Figure 3A

U33

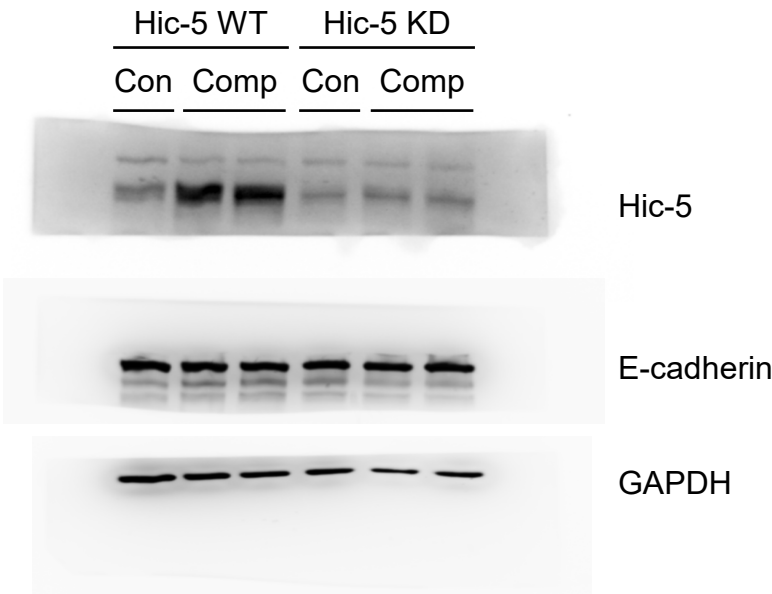

U28

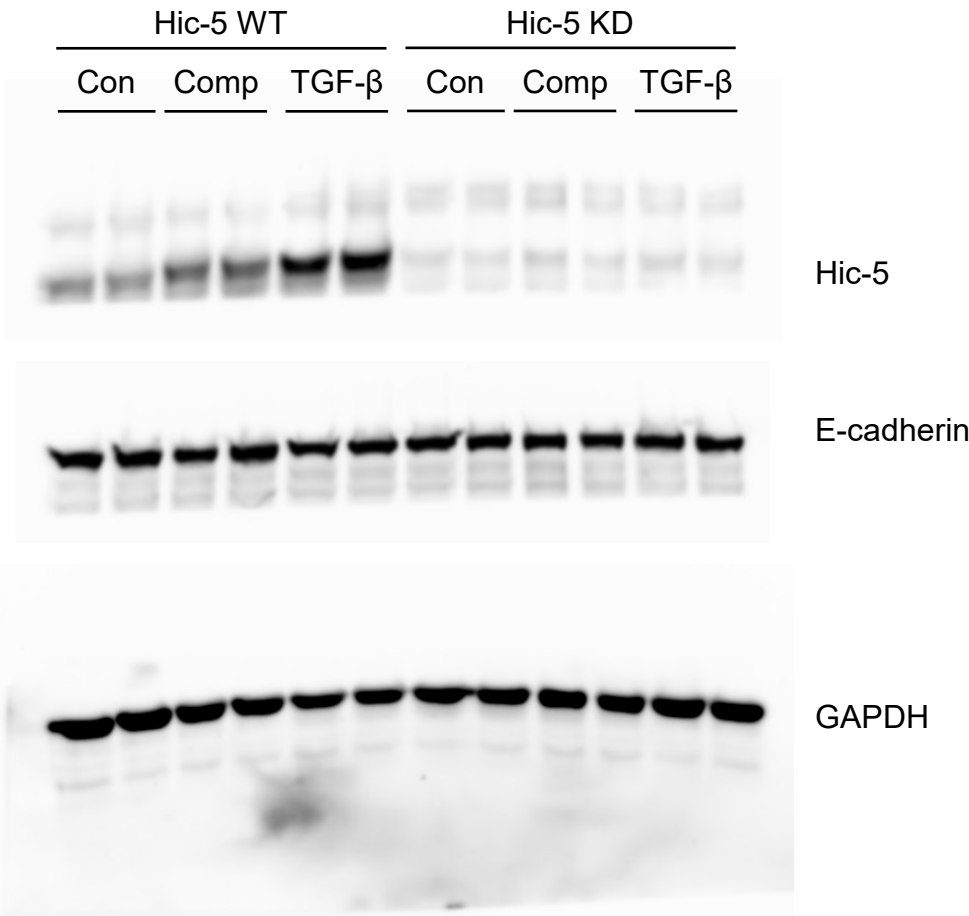

Supplement: Supplementary file 4 — Source Data [file 41467_2025_67210_MOESM4_ESM.zip › Source Data_uncropped images.pdf]
